# Combined Radiocarpal Dislocation and Forearm Joint Injuries: A Proposed Modification of the Locker-Based Classification System

**DOI:** 10.7759/cureus.57369

**Published:** 2024-04-01

**Authors:** Rastislav Burda, Ildikó Morochovičová, Róbert Křemen

**Affiliations:** 1 Department of Trauma Surgery, Faculty of Medicine Pavol Jozef Šafárik University in Košice, Kosice, SVK; 2 Department of Trauma Surgery, Louis Pasteur University Hospital in Košice, Kosice, SVK; 3 Department of Physiotherapy, Faculty of Medicine Pavol Jozef Šafárik University in Košice, Košice, SVK; 4 Department of Physiotherapy, Louis Pasteur University Hospital in Košice, Kosice, SVK

**Keywords:** radiocarpal dislocation, elbow dislocation, comprehensive locker-based classification system, modification, combination

## Abstract

The comprehensive locker-based classification system brought revolutionary insight into the treatment of often misdiagnosed forearm joint injuries. The authors of the classification scheme subsequently described a combination of simple elbow dislocations and forearm joint injuries (two- and three-locker injuries), but to date, no review of the literature on combined radiocarpal dislocation and forearm joint injuries has been undertaken. The combination of radiocarpal dislocation and forearm joint injury is a rare traumatic pattern, usually related to high-energy trauma. The aim of this study was to confirm the possible occurrence of a forearm joint injury and radiocarpal dislocation. We performed a systematic review of the existing literature, including case reports, to find combinations of radiocarpal dislocation and forearm joint injury. Only one case report was found. Based on the results of our search and the literature review, we recommend modifying the comprehensive locker-based classification system by adding injury patterns of combined forearm joint and neighboring joint injuries.

## Introduction

The comprehensive locker-based classification system is based on the current understanding of anatomy and the functions of the forearm, which were presented by Soubeyrand et al. and Dumontier [1,2].

The forearm is understood to be a triarticular complex structure (a three-locker system) that functions as a full-fledged entity. The three forearm radioulnar joints (proximal, middle, and distal) work together to provide stability, mobility, and load transfer. Two of them are anatomical joints: the proximal radioulnar joint (PRUJ) and the distal radioulnar joint (DRUJ). The third, the middle radioulnar joint, is a functional joint. The joints may be locked, absent, or unstable, with varying insufficiency combinations recognized in many already described forearm fracture-dislocation patterns [3].

After a meticulous search of the relevant literature, Artiaco et al. [4] included all types of dislocation and fracture-dislocation of the forearm joint that were reported in the literature in the comprehensive locker-based classification system. The system simplified the classification and treatment of fracture-dislocations of the forearm joints. It identifies 13 combinations of forearm joint fracture-dislocation (two- and three-locker injuries). This comprehensive classification system revolutionized the treatment of these often-misdiagnosed injuries. Its simplicity and memorability brought the use of eponyms in clinical practice to an end. Three-locker injuries are rare, often overlooked, and frequently underestimated. Most of the injury patterns are described in the literature; however, there is a limited amount of information available about their etiology.

According to further research by Artiaco et al., simple elbow dislocation combined with a forearm joint injury involving two or three lockers is described in the literature. Yet, there are a mere 15 documented cases of such injuries, and all the articles are only case reports [5].

In our clinical practice, we encountered a case that had not yet been documented in any publication. This case presented a combination of radiocarpal dislocation and forearm joint injury.

## Case presentation

Case description

**Figure 1:**
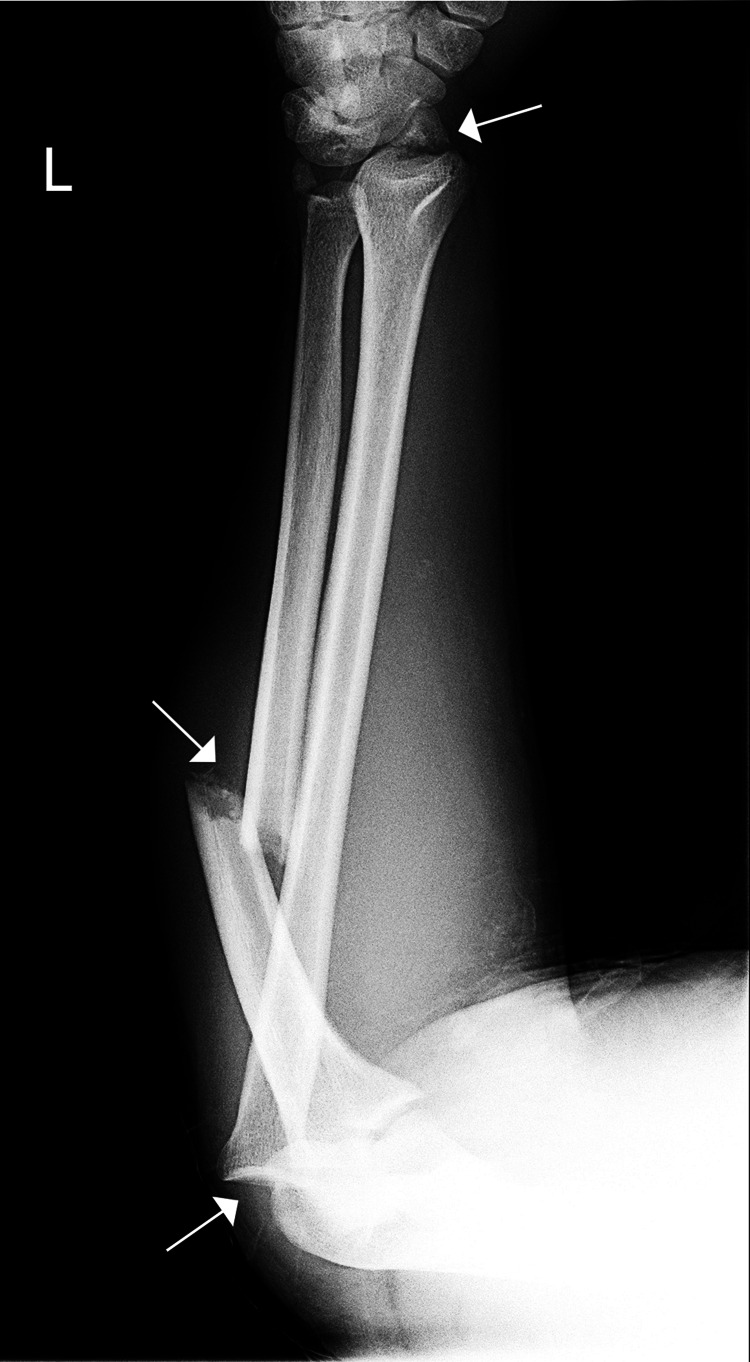
Initial X-rays of the forearm Proximal radioulnar dislocation (lateral radial head dislocation), an ulnar shaft fracture and radiocarpal dislocation.

**Figure 2:**
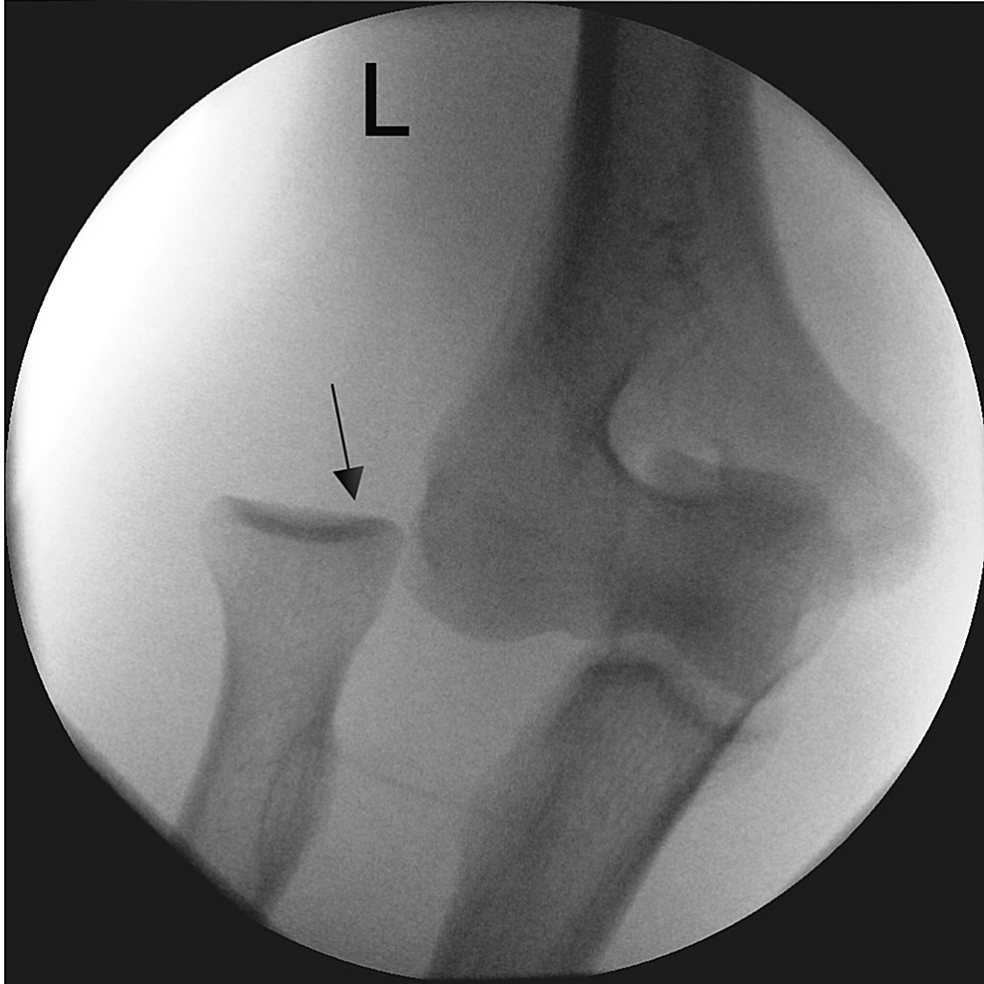
Perioperative X-rays: lateral dislocation of the radial head Lateral dislocation of the radial head combined with a concomitant injury is rarely seen.

**Figure 3:**
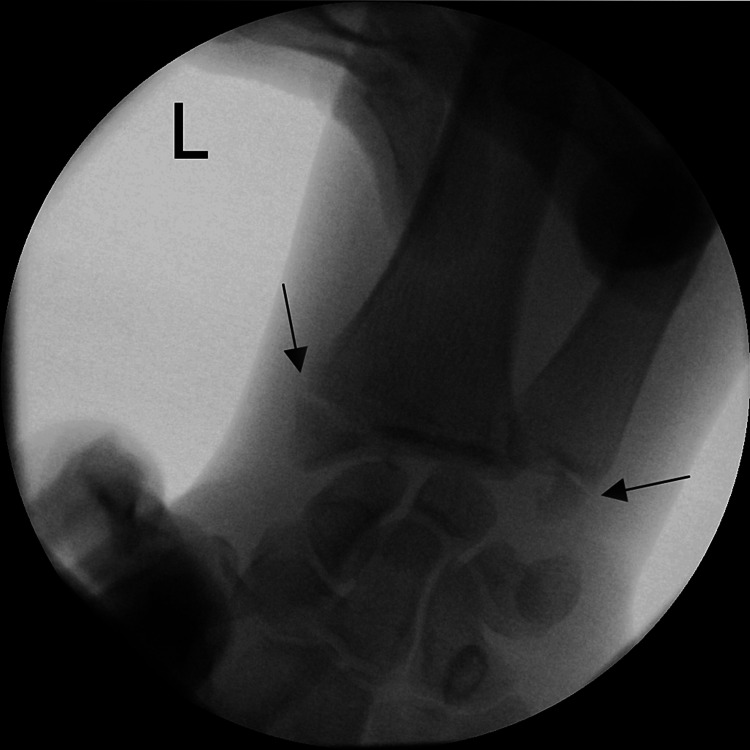
Perioperative X-rays of the wrist, posterior-anterior view Visible fracture of both radial and ulnar styloid processes, but radiocarpal dislocation is not clearly visible in this view.

**Figure 4:**
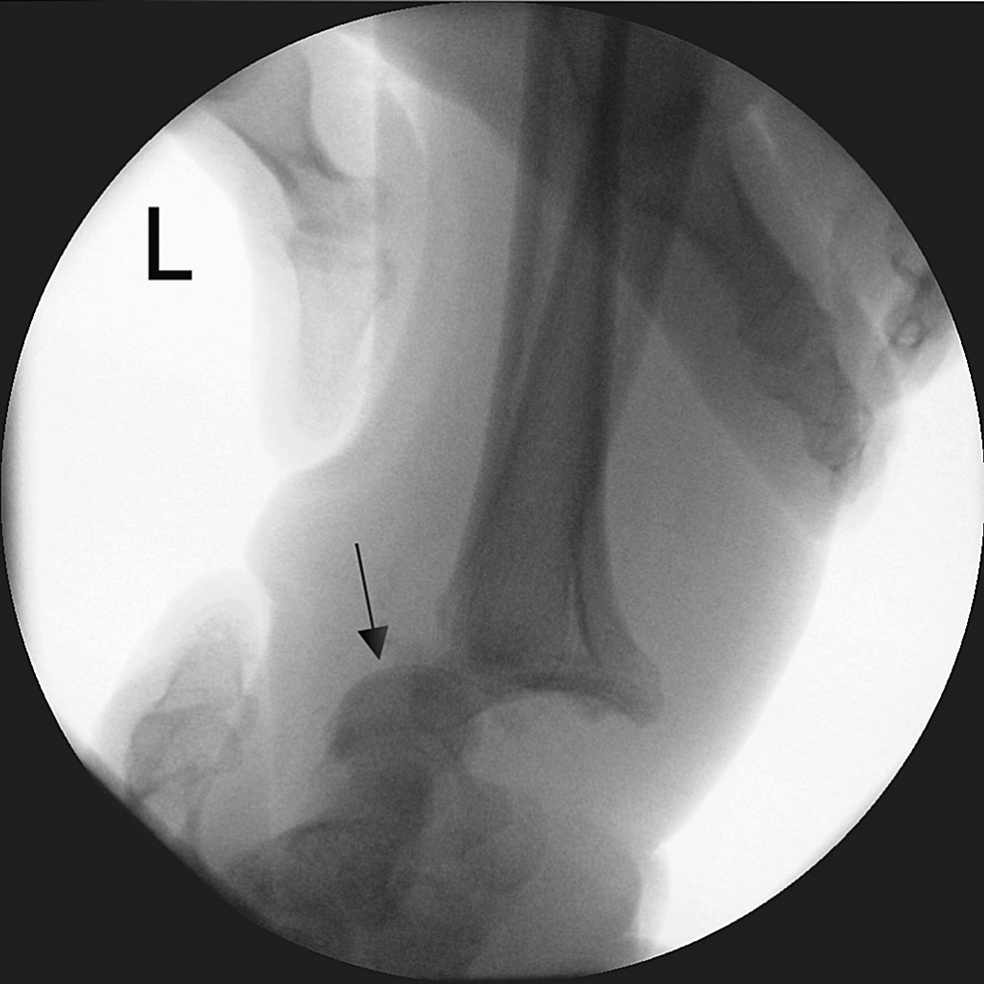
Perioperative X-rays of the wrist The lateral view shows visible radiocarpal dislocation.

**Figure 5:**
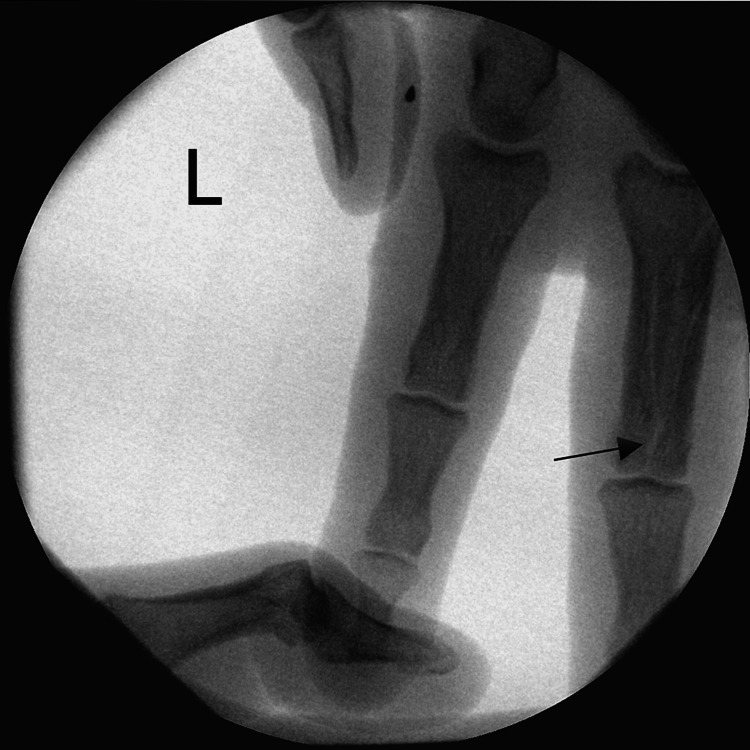
Longitudinal fracture of the proximal phalange on the third finger on the same side The unusual fracture pattern (longitudinal fracture of the phalange) confirms force acting on the long axis of the forearm.

A 24-year-old man presented to our institution with a history of falling onto an outstretched hand and the subsequent sudden bending of the wrist after a fall from a sports motorcycle. Evaluation in the emergency department revealed proximal radioulnar joint dislocation with lateral radial head dislocation and an ulnar shaft fracture associated with radiocarpal dislocation (Figures 1-5).

**Figure 6:**
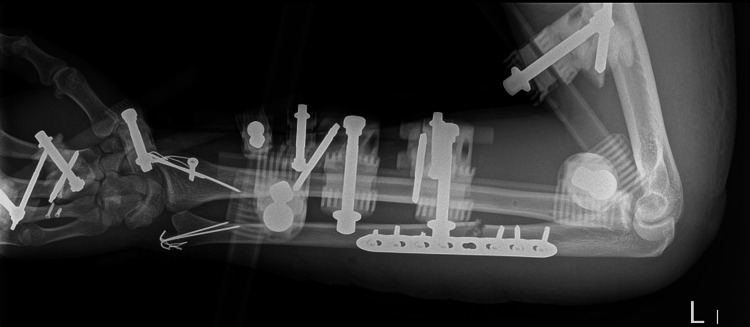
Open reduction and internal fixation of both forearm joints and additional fixation with external fixator: elbow and wrist bridging construction Close reduction of dislocation of the radial head and internal fixation of an ulnar shaft fracture with locking compression plate and tension band wiring for radial and ulnar styloid fracture. According to our suggestion, it should be classified as 0(1.2IU)1.

The patient underwent urgent surgery. The fracture of the diaphysis of the ulna was fixed with a locking compression plate, and after reaching the anatomical reduction of the ulna, a spontaneous closed reduction of the radial head occurred. The radiocarpal dislocation was closed reduced, and subsequently, the radial and ulnar styloid fractures were fixed by tension band wiring. In addition, the elbow and wrist joints were bridged by an external fixator for three weeks (Figure 6), while the wrist was immobilized for the next three weeks with a plaster cast. The subsequent treatment was without any complications. Evaluation at the 12-month post-injury revealed a full range of motion in the elbow and wrist without any instability.

Literature search

We decided to perform a systematic search for combinations of radiocarpal dislocation and fracture-dislocation of the forearm joint. This is a rare clinical condition that occurs due to high-energy trauma, and it has never been investigated as a combined traumatic injury.

Materials and Methods

The authors performed a systematic review of the available literature on the major conditions involving radiocarpal dislocation and fracture-dislocation of the forearm joint. Based on the review of the available literature, the descriptions contained within Artiaco et al.’s classification scheme, and the case documented by the authors of this article, we designed a modification for Artiaco et al.’s comprehensive classification scheme.

Literature Search

In accordance with the Preferred Reporting Items for Systematic Reviews and Meta-Analyses (PRISMA) guidelines [6], on August 4, 2023, we conducted a comprehensive electronic search through PubMed. The following keywords were used in a PubMed search: (radiocarpal dislocation) AND (((galeazzi) OR (monteggia) OR (essex-lopresti) OR (forearm) OR (((proximal) OR (distal)) AND (radio ulnar))). We used the same keywords for the PubMed search as Artiaco used in his work, but the phrase “elbow dislocation” was replaced with “radiocarpal dislocation“ [5].

The selection was limited to human subjects. Clinical studies, clinical trials, and case reports written in English, German, and French were included. All articles responding to the searched keywords were reviewed, with no date set as a restriction. All articles related to pediatric and adolescent patients (under 18 years of age), papers reporting isolated forearm joint injuries, and works without the combination of forearm joint injuries and radiocarpal dislocation were excluded.

Data Extraction

Two authors (RB and RK) independently and repeatedly screened the titles and abstracts of all relevant articles that contained the keywords. Reference lists from the selected articles were used for an additional search. The selected records were further inspected, and the full-text articles were carefully scrutinized.

Results

**Figure 7:**
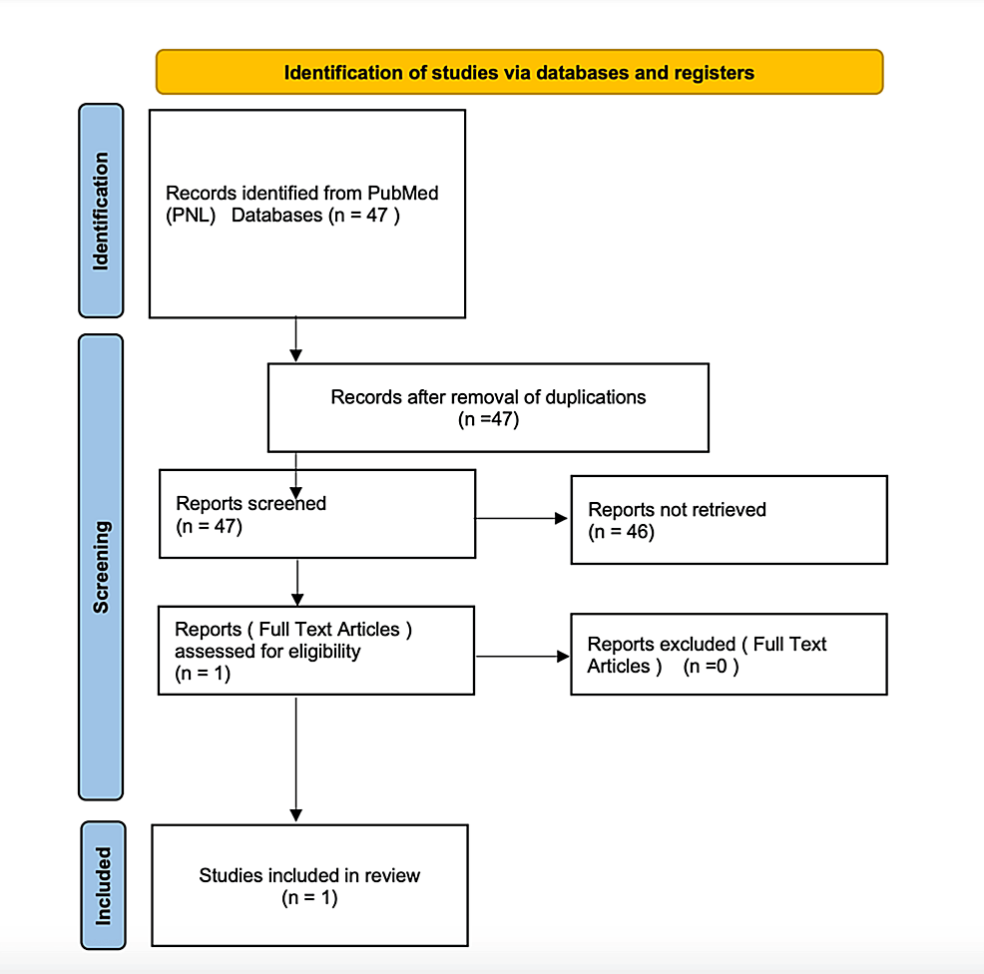
Flow diagram of the systematic review of the literature

A total of 47 studies were identified in the primary literature search. A flow diagram of the systematic review is shown in Figure 7.

The identified 47 articles were further analyzed. As a result, 46 articles were excluded because their title or abstract did not meet the designated inclusion criteria (off-topic articles that did not mention a combination of radiocarpal dislocation and forearm joint injury). Only one article complied with all inclusion criteria, which is why meta-analysis was deemed inappropriate. A narrative approach was selected instead. The article was a case report in which Stoffelen described volar DRUJ dislocation combined with trans-styloid radiocarpal dislocation. In this case, the radial styloid was fixed by an open reduction, and the DRUJ dislocation was revised [7].

## Discussion

Artiaco et al. [4] included all types of fracture-dislocation of the forearm joint reported in the literature in the comprehensive locker-based classification system, which describes 13 combinations of forearm joint fracture-dislocation (two- and three-locker injuries). The initial classification system depicted no combined injuries (forearm joint injuries and concomitant injuries of adjacent joints), but the author later added a description of a simple elbow dislocation and forearm joint injury.

Simple elbow dislocations combined with forearm joint injuries involving two or three lockers are highly uncommon injuries, according to their description in the literature. Only 15 cases of such injuries have been documented, and the articles are only case reports [5].

We found similarities between the injuries reported in the case found by our search and our case. The rarity of the mentioned combination of injuries led us to conduct a manual search for possible combinations of forearm joint injuries and wrist injuries. Only two records were retrieved by the manual search. The two articles described different combinations of wrist injuries and complex forearm joint injuries. Descriptions of these case reports are provided in Table 1.

**Table 1:** Summary of articles describing a combination of forearm joint injury and wrist injury

Author	Wrist injury pattern	Accompanying forearm joint injury
Stoffelen et al., 1996 [7]	Trans-styloid radiocarpal dislocation	Volar distal radioulnar joint dislocation (2.I.3)
Gul et al., 2007 [8]	Dislocation of the lunate bone	Monteggia fracture (1.2IU)
Stahl and Freiman, 1999 [9]	Scaphoid fracture	Monteggia fracture (1.2 IU)

Gul et al. described the combination of a Monteggia fracture and a volar dislocation of the lunate bone. Manual open reduction of the lunate bone was carried out, and the wrist was temporarily stabilized with K wires [8]. Stahl and Freiman described simultaneous combinations of Monteggia fractures and scaphoid fractures [9].

Wrist injuries, combined with forearm joint injuries, are extremely rare. Ultimately, we found only three published cases of such traumatic injuries.

**Figure 8:**
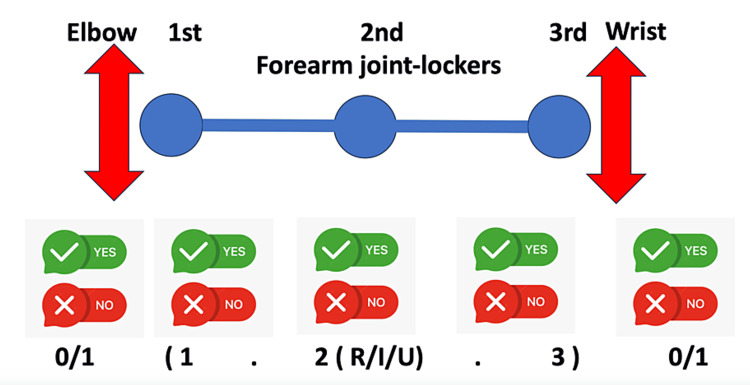
Proposal for the modification of the comprehensive locker-based classification system The injuries should be designated as, for example, 0/1 (1.2(R/I/U).3) 0/1 R: radius, I: interosseous membrane, U: ulna Image Credit: Rastislav Burda

These facts led us to propose a modification of the comprehensive locker-based classification system. We suggest adding injuries of neighboring joints (elbow and wrist) to the scheme because, in the treatment of forearm injuries, we should increase the focus on the neighboring joints due to the possibility of a combination of these injuries (Figure 8).

The proposed modification of the classification system should lead to more precise descriptions of combined injury patterns.

From a practical standpoint, when it comes to the designation of the injuries, we recommend bracketing the scoring of the forearm joint injury (Artiaco et al.’s scoring) and adding either the number 0 or 1 before or after the bracket to indicate whether an injury to the adjacent joint (elbow, wrist) is present.

## Conclusions

Combined forearm joint and elbow or wrist injuries can sometimes be observed in clinical practice. Wrist and elbow joints must always be examined in clinical and radiological investigations of forearm injuries due to the possibility of neighboring joints being involved.

Based on our findings, the modification of the comprehensive locker-based classification system and the addition of patterns of combined forearm and neighboring joint injuries are justifiable.
